# Testing the organizational theory of innovation implementation effectiveness in a community pharmacy medication management program: a hurdle regression analysis

**DOI:** 10.1186/s13012-018-0799-5

**Published:** 2018-07-31

**Authors:** Kea Turner, Justin G. Trogdon, Morris Weinberger, Angela M. Stover, Stefanie Ferreri, Joel F. Farley, Neepa Ray, Michael Patti, Chelsea Renfro, Christopher M. Shea

**Affiliations:** 1 Department of Health Policy and Management, Gillings School of Global Public Health, The University of North Carolina at Chapel Hill, 135 Dauer Drive, Chapel Hill, NC 27599-7411 USA; 2 Division of Practice Advancement and Clinical Education, Eshelman School of Pharmacy, The University of North Carolina at Chapel Hill, 115B Beard Hall, Chapel Hill, NC 27599-7411 USA; 3 Department of Pharmaceutical Care and Health Systems, College of Pharmacy, University of Minnesota, 308 Harvard Street SE, Minneapolis, MN 55455 USA; 4 Center for Medication Optimization through Practice and Policy, Eshelman School of Pharmacy, The University of North Carolina at Chapel Hill, 2400 Kerr Hall, Chapel Hill, NC 27599-7411 USA; 5 Department of Clinical Pharmacy and Translational Science, University of Tennessee Health Science Center, 881 Madison Avenue, Memphis, TN 38163 USA; 6 Department of Health Policy and Management, Gillings School of Global Public Health, The University of North Carolina at Chapel Hill, 1103E McGavran-Greenberg, 135 Dauer Drive, Chapel Hill, NC 27599-7411 USA

**Keywords:** Implementation climate, Innovation-values fit, Community pharmacy, Medication management, Organizational theory

## Abstract

**Background:**

Many state Medicaid programs are implementing pharmacist-led medication management programs to improve outcomes for high-risk beneficiaries. There are a limited number of studies examining implementation of these programs, making it difficult to assess why program outcomes might vary across organizations. To address this, we tested the applicability of the organizational theory of innovation implementation effectiveness to examine implementation of a community pharmacy Medicaid medication management program.

**Methods:**

We used a hurdle regression model to examine whether organizational determinants, such as implementation climate and innovation-values fit, were associated with effective implementation. We defined effective implementation in two ways: implementation versus non-implementation and program reach (i.e., the proportion of the target population that received the intervention). Data sources included an implementation survey administered to participating community pharmacies and administrative data.

**Results:**

The findings suggest that implementation climate is positively and significantly associated with implementation versus non-implementation (AME = 2.65, *p* < 0.001) and with program reach (AME = 5.05, *p* = 0.001). Similarly, the results suggest that innovation-values fit is positively and significantly associated with implementation (AME = 2.17, *p* = 0.037) and program reach (AME = 11.79, *p* < 0.001). Some structural characteristics, such as having a clinical pharmacist on staff, were significant predictors of implementation and program reach whereas other characteristics, such as pharmacy type or prescription volume, were not.

**Conclusions:**

Our study supported the use of the organizational theory of innovation implementation effectiveness to identify organizational determinants that are associated with effective implementation (e.g., implementation climate and innovation-values fit). Unlike broader environmental factors or structural characteristics (e.g., pharmacy type), implementation climate and innovation-values fit are modifiable factors and can be targeted through intervention—a finding that is important for community pharmacy practice. Additional research is needed to determine what implementation strategies can be used by community pharmacy leaders and practitioners to develop a positive implementation climate and innovation-values fit for medication management programs.

## Background

Many state Medicaid programs have expanded enrollment eligibility under the Affordable Care Act, making Medicaid the largest health insurance program in the USA [1, 2]. Medicaid spending is largely driven by a small subset of high-risk patients; 5 % of Medicaid beneficiaries account for almost half of Medicaid expenditures [3]. This small subset of beneficiaries is disproportionately impacted by chronic conditions, such as diabetes and asthma, and the co-occurrence of difficult-to-treat conditions (e.g., substance use and mental health conditions) [3]. To improve chronic disease management, several Medicaid programs have implemented medication management programs in partnership with pharmacists [4–6].

Pharmacist-led medication management programs have improved patients’ medication adherence and therapeutic outcomes (e.g., blood pressure, hemoglobin A1C) while reducing healthcare costs [7–10]. However, researchers have had difficulty attributing changes in patient outcomes to specific program features due to the wide variability in medication management programs [4, 11]. In the Medicare Part D Medication Therapy Management (MTM) program, for example, researchers have noted that medication services are delivered in a variety of settings (e.g., call centers, outpatient care) and formats (e.g., in-person vs. phone) [11]. Similar challenges exist in Medicaid medication management programs—programs vary in patient eligibility criteria, the services provided, and the setting of service delivery [4].

In addition to program design variability, there are a limited number of studies examining implementation of pharmacist-led medication management programs, making it difficult to assess why program outcomes might vary across organizations. Many of the studies that have examined organizational determinants of implementation effectiveness in pharmacist-led medication management programs have been qualitative, limiting their generalizability, or have not been guided by a theory, making it difficult to interpret the findings. Past studies have identified factors, such as organizational structure (e.g., staff size), leadership support, and financial resource availability [12–16], but not applied theory to demonstrate how these factors work in concert to produce effective implementation. Thus, this study will test the applicability of the organizational theory of innovation implementation effectiveness to examine implementation of a community pharmacy Medicaid medication management program.

### Conceptual framework

**Fig. 1:**
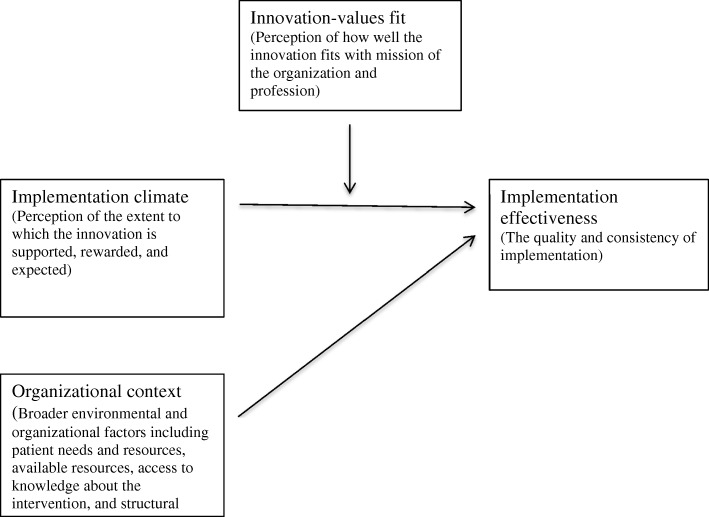
The impact of implementation climate and innovation-values fit on implementation effectiveness

Implementation theories have been developed to identify the organizational factors and underlying relationships that are hypothesized to influence effective implementation (i.e., the quality and consistency of implementation) [17, 18]. The organizational theory of innovation implementation effectiveness was designed for complex innovations like medication management programs, which often require coordinated use by multiple individuals to be effective [18–20]. This theory posits that effective implementation is driven by an organization’s implementation climate and the fit between the innovation and organization values (Fig. 1) [18–20]. For example, a community pharmacy might develop formal policies to support implementation of a medication management program such as employee training or reward and recognition systems. The collective influence of the pharmacy’s implementation policies, in turn, affects employees’ shared perceptions about the extent to which the medication management program is rewarded, supported, and expected (implementation climate) [18–20]. Positive perceptions about implementation climate are likely to increase employees’ acceptance of medication management programs, increasing the likelihood that pharmacy staff will appropriately implement medication management programs (i.e., as the pharmacy intended for it to be implemented) and ultimately increase implementation effectiveness. Therefore, we hypothesize that positive perceptions about implementation climate will be positively associated with implementation effectiveness (H1).

The organizational theory of innovation implementation effectiveness maintains that innovation-values fit also affects implementation effectiveness [18–20]. In this specific case, innovation-values fit refers to pharmacy employees’ perceptions about how well medication management programs align with the values of the pharmacy and the pharmacy profession. Innovation-values fit is believed to affect implementation effectiveness both directly and indirectly. If pharmacy employees perceive that medication management programs do not align with the pharmacy’s values, the employee may be less committed to implementation and exert less effort towards ensuring effective implementation [18]. Innovation-values fit is also likely to impact the relationship between implementation climate and implementation effectiveness [18–20]. Since innovation-values fit affects commitment, the impact of implementation climate on implementation effectiveness will be amplified by positive, and weakened by negative, perceptions about innovation-values fit. Thus, we hypothesize that positive perceptions about innovation-values fit will directly and positively affect implementation effectiveness (H2) and moderate the relationship between implementation climate and implementation effectiveness (H3).

Implementation effectiveness is also likely to be affected by broader environmental and organizational factors (e.g., organizational context), such as patient needs and resources, available resources, access to knowledge about the intervention, and structural characteristics [21]. For example, community pharmacies that serve a higher proportion of high-risk patients may be better at implementing innovations for high-risk populations. Additionally, pharmacies in rural locations may be better at implementing innovations for high-risk populations since residents in rural areas have higher rates of chronic illness [22]. Implementation effectiveness is also likely to be positively influenced by a pharmacy’s available resources, such as amount of staff and training of staff, and access to knowledge about medication management programs (e.g., experience implementing similar interventions). Conversely, certain structural characteristics may negatively affect implementation effectiveness. For example, pharmacies that have opened recently may not have as strong of ties with patients as pharmacies that have been in operation for many years (e.g., the liability of newness hypothesis) [23]. Similarly, larger pharmacies may be impeded by a more formal organizational structure, which can negatively impact innovation implementation [24]. For instance, managers of independently owned pharmacies may have greater decisional autonomy because there is less formalization in the organization and, as a result, be better able to support implementation of medication management programs. Therefore, we hypothesize that four contextual factors, patient needs and resources, available resources, access to knowledge about the intervention, and structural characteristics, will affect implementation effectiveness (H4).

## Methods

### Study design

We used a cross-sectional design examining implementation of a community pharmacy Medicaid medication management program during the program year of 2016. The unit of analysis was at the pharmacy level.

### Intervention description

The community pharmacy enhanced services network (CPESN^SM^) is a national network of community pharmacies that offer medication management services [25]. This study examines the North Carolina CPESN (NC-CPESN), the pioneer site for CPESN [6]. NC-CPESN was launched in 2014 by the Community Care of North Carolina (CCNC)—the primary case management provider for NC Medicaid beneficiaries [6, 26]. NC-CPESN is voluntary and allows any community pharmacy if the following requirements are met: (1) provide certain medication management services, (2) be responsible for the outcomes of a defined patient population through value-based payment, and (3) tailor services based on patients’ risk score. One of the key services required for reimbursement is a comprehensive medication review (CMR) to identify opportunities for improving medication management and reduce risk of medication problems. Patients include Medicaid, Medicare, and NC Health Choice beneficiaries, as well as dual-eligible patients. The payment model is a per-member per-month payment model based on patient’s risk score (described below) and the pharmacy’s performance score on a series of measures including medication adherence, total cost of care, hospital admission rate, and emergency department admissions [27].

### Study population

The study population included community pharmacies that participated in either the first year or the second year of the 3-year NC-CPESN program (September 2014–August 2017); pharmacies that joined in the third year were excluded from the analysis because they had little-to-no experience with implementation at the time of the survey (described below).

### Data sources

In fall 2016, we administered a paper-based survey to community pharmacies that participated in either the first or second year of the NC-CPESN program. The survey assessed pharmacies’ structural characteristics, experience with NC-CPESN, and perceptions about implementation (e.g., implementation climate, innovation-values fit). A copy of the survey has been published elsewhere [28]. A committee of researchers and community pharmacy practitioners (*n* = 25) reviewed the survey items’ content, readability, and formatting. The survey questions were also piloted in a small group of community pharmacists (*n* = 5) who were identified as experts by the committee based on their job tenure and reputation in the field of community pharmacy. From the committee review and the initial pilot test, we received similar types of feedback and felt that further pilot testing was not needed. The survey was mailed to participating pharmacies along with other NC-CPESN program materials to increase the response rate. Pharmacies also received three email reminders at ~ 2, 4, and 8 weeks after the survey was mailed. Within the pharmacy, employees that were intended users of NC-CPESN (e.g., pharmacists, pharmacy technicians, and administrative staff) completed the survey. We did not include supporters of NC-CPESN (e.g., pharmacy owners who supported the intervention through policies and resources but did not directly implement the intervention) since their actions indirectly rather than directly affect implementation, a decision that is consistent with other implementation studies [19, 29]. We had more than one respondent per pharmacy; therefore, the responses were aggregated to the pharmacy level (description below). We received surveys from 191 of 268 pharmacies (71.3% response rate). Participants provided written consent. The Institutional Review Board of the University of North Carolina at Chapel Hill approved this study (IRB # 17-1304).

In addition, we used 2016 NC-CPESN program administrative data and 2016 county health ranking data [30]. Program administrative data provided information on the number of high-risk patients attributed to each pharmacy, patient demographics, and the number of CMRs that were delivered. County health ranking data included county-level measures of clinical (e.g., healthcare access) and social (e.g., insurance status) factors that might affect the pharmacy’s implementation of NC-CPESN [30]. The operationalization of these measures is described below.

### Dependent variables

We used two variables to measure implementation effectiveness—one indicator for implementation versus non-implementation and one indicator for program reach.

#### Implementation of a CMR for high-risk patients

Based upon whether a pharmacy implemented a CMR on any attributed high-risk patient, we divided the sample into implementers (e.g., ≥ 1 CMR for an attributed high-risk patient) and non-implementers (e.g., no CMR for any attributed high-risk patients) during the program quarter Nov. 2016–Jan. 2017. We chose this quarter because there were no changes to the intervention (e.g., intervention requirements or payment model) during this or the previous quarter. High risk was defined as having a care triage score ≥ 75. Care triage score is a proprietary measure used by CCNC to estimate a patient’s risk for hospitalization and includes variables such as the number of chronic conditions a patient has and the type of medication the patient is taking. Patients with care triage scores > 75 are considered a priority population for CCNC. Patients are defined as attributed to a pharmacy if they filled at least one chronic medication within the last 90 days and ≥ 80% of their medications for at least two of 3 months within the quarter. Patients also had to be eligible for Medicaid or Medicare for at least two of the 3 months within the quarter. The patient attribution process is done on a monthly basis. On average, in any given month, about 100,000 NC residents are attributed to NC-CPESN pharmacies [27].

#### Proportion of high-risk patients receiving a CMR

We measured implementation effectiveness to assess the reach of the intervention among attributed high-risk patients. We calculated the number of attributed high-risk patients receiving a CMR divided by the number of high-risk, attributed patients per pharmacy during the program quarter Nov. 2016–Jan. 2017.

### Independent variables

#### Implementation climate

Implementation climate was defined using four survey items assessing the extent to which NC-CPESN was supported, rewarded, and expected within the pharmacy (e.g., “Our pharmacy allocates sufficient time to delivering enhanced pharmacy services” and “Our pharmacy devotes adequate resources to implementing enhanced pharmacy services”) [18–20]. The survey items were adapted for a pharmacy setting from a scale validated in an oncology setting [31, 32]. The questions included group rather than individual referents, which is recommended when assessing organizational-level outcomes such as implementation climate [29]. Each item was measured on 5-point Likert scale ranging from 0 (strongly disagree) to 4 (strongly agree). The survey items were summed for individual staff members who worked directly on implementation (i.e., innovation users), and a mean was calculated to produce a pharmacy-level measure. Higher values of the score corresponded with positive perceptions of implementation climate.

#### Innovation-values fit

Innovation-values fit was defined using four survey items assessing staff perceptions about the extent to which NC-CPESN fit with the values of the pharmacy (e.g., “Delivering enhanced pharmacy services is consistent with providing the best care possible for our patients”) and of the pharmacy profession (“Delivering enhanced pharmacy services is important for advancing the field of pharmacy”) [18–20]. To identify “high-intensity” values, or values that are highly important to pharmacy staff, we obtained pharmacy practitioner input during the survey pilot (described above) [18–20]. For example, practitioners described the importance of improving the quality of services across all community pharmacies, not just the quality of services within their own pharmacy. As a result, practitioners described valuing interventions that would advance the field of community pharmacy as a whole. To address this, we included a survey item to assess perceptions about whether NC-CPESN was advancing the field of pharmacy. As with implementation climate, the innovation-values fit questions were group-referenced, measured on the same 5-point Likert scale, aggregated from individual responses to produce a pharmacy-level mean, and ordered so that higher scores corresponded to positive perceptions.

#### Other independent variables

Patients’ needs and resources were measured by rural location, clinical factors, social factors, 340B participation, and proportion of high-risk patients. Rural location was defined as a binary variable (e.g., urban, rural) using a zip code approximation of the rural-urban commuting area codes. Clinical factors were defined using a pre-existing, county-level composite measure of access to care items (e.g., primary care provider ratio, uninsured rate) and quality of care items (e.g., preventable hospital stays, diabetes monitoring) ranging from 0 to 100 [30]. Social factors were defined using a pre-existing, county-level composite measure of items such as education, employment, uninsured, and income ranging from 0 to 100 [30]. The clinical and social factor scales were recoded so that higher values on the scale were associated with better patient outcomes. Participation in 340B Drug Pricing Program was measured as a binary variable. The 340B Drug Pricing Program is a federal program that requires drug manufacturers to provide outpatient drugs to eligible healthcare organizations (e.g., safety net providers) at a discounted rate [33]. Community pharmacies can participate in this program by dispensing 340B drugs through a contract with eligible healthcare organizations. Proportion of high-risk patients was defined as the number of attributed high-risk patients divided by the number of attributed patients per pharmacy over a program quarter.

#### Available resources

Available resources were measured by three variables: the presence of a clinical pharmacist (binary), total number of full- and part-time staff (e.g., pharmacists, pharmacy technicians, administrative staff), and the presence of pharmacy students or residents in the past month (binary). A clinical pharmacist is defined as a pharmacist whose role focuses not only on dispensing medication but also on the clinical care of patients, such as optimizing patients’ medication regimens and providing health education and preventive health services [34]. In community pharmacies, a clinical pharmacist typically has some or all of their time devoted to activities outside of dispensing medications, such as delivering medication management services [35]. The role of clinical pharmacist, such as the type of clinical services offered within the pharmacy, as well as the amount of time devoted to non-dispensing activities, varies, however, across community pharmacy settings.

#### Access to knowledge about the intervention

Access to knowledge about the intervention was measured in three ways: (1) experience with NC-CPESN, defined as the number of months the pharmacy was enrolled in NC-CPESN; (2) past performance with NC-CPESN, measured using a lagged dependent variable (e.g., proportion of CMRs completed per high-risk patients) for the previous program quarter (Aug–Oct 2016); and (3) participation in Medicare Part D MTM (binary).

#### Structural characteristics

Structural characteristics were assessed by three variables. First, independent ownership was a binary variable: single- and multiple- independent pharmacies versus chain, outpatient, and federally qualified health center (FQHC) pharmacies. Independently owned pharmacies include single-independent pharmacies (i.e., an owner owns one pharmacy) and multiple independent pharmacies (i.e., an owner owns multiple pharmacies). Chain pharmacies include pharmacies owned by a publically traded company. Outpatient pharmacies are pharmacies operated by a health system and located within an outpatient healthcare setting (e.g., primary care office), and FQHC pharmacies are operated by and located within a FQHC. We used a binary variable due to small sample sizes within the chain and outpatient pharmacy categories. Second, prescription volume was dichotomized as low (< 2000 prescriptions/week) versus high (≥ 2000 prescriptions/week). The criterion for low volume was selected based on input of community pharmacy practitioners and researchers. Third, established pharmacies were those that had been in operation for more than 20 years. Similarly, the threshold of 20 years as the criterion for established pharmacy was selected based on input from community pharmacy practitioners and researchers.

### Statistical analysis

#### Descriptive statistics

Frequencies and percentages were used to describe the study population. We conducted bivariate analyses to compare the sample characteristics between implementers (completed ≥ 1 CMR during the program quarter for high-risk patients) and non-implementers (no completed CMR during the program quarter for high-risk patients).

#### Exploratory factor analyses

To determine if implementation climate and innovation-values survey items could be used as distinct variables, we conducted three analyses. First, we examined pairwise correlations among the items and conducted a Bartlett’s test of sphericity and a Kaiser-Meyer-Olkin (KMO) test. Second, we defined the number of initial factors using principal component analysis and rotated the factors using orthogonal varimax rotation to improve interpretability. Finally, we confirmed the number of extracted factors using two decision rules: (1) the number of eigenvalues > 1.0, and (2) the number of eigenvalues from the factor analysis that were larger than the eigenvalues from randomly generated data (e.g., parallel analysis test). We also assessed the internal consistency of the two scales using Cronbach’s coefficient alpha. To ensure the results were not overly sensitive to the method of factor extraction, we conducted a sensitivity analysis by running a common factor analysis using principal axis factoring and did not find differences in the results. We also compared the results of the exploratory factor analyses by staff roles within pharmacies (e.g., pharmacists, pharmacy technicians, and administrative staff) to determine if results from different staff types could be aggregated to the pharmacy level. Factor analyses did not differ by subgroup, suggesting that aggregating subgroups was appropriate.

#### Hurdle regression model

Hurdle regression is a two-equation model for count data: one equation determines the likelihood of an outcome (e.g., whether a pharmacy implemented a CMR) and the other examines the positive outcomes (e.g., how many CMRs were delivered to high-risk patients) [36, 37]. We used a hurdle regression to model both of these processes and to account for an excess of zeroes in the dependent variable (40.8% of the sample had zero implementation in the program quarter). For the first stage, we used a logistic regression to determine the probability of a pharmacy implementing a CMR for a high-risk patient (e.g., implementer versus non-implementer). For the second stage, we used a zero-truncated negative binomial model to determine how many CMRs were delivered to high-risk patients (e.g., program reach). A negative binomial model was selected over a Poisson model to account for over-dispersion in the data (i.e., the variance was larger than the mean). For the negative binomial model, we treated the denominator (i.e., number of high-risk patients) as the exposure to adjust for differences in the opportunity available to deliver the intervention and assumed the unobserved heterogeneity was gamma distributed (i.e., NB2 model). We compared this model with a zero-inflated negative binomial, which is another two-equation model for count data; we did not find differences in the results. Therefore, we used the hurdle regression.

In the hurdle regression, we included the key variables of interest (e.g., implementation climate, innovation-values fit, and an interaction of the two) and control variables selected a priori (e.g., patient needs and resources, available resources). We assessed the goodness of fit for the interaction term in both stages of the model since interpretation of marginal effects on interaction terms can be complicated in non-linear models [38]. Since the interaction term improved fit in both stages, we included the term. To model the impact of the interaction term, we plotted the marginal effect of innovation-values fit over representative values of implementation climate score for both of the equations. One control variable, past performance with NC-CPESN, was a lagged dependent variable, which can cause biased coefficients if the data generating process is non-stationary [39]. Using the Harris-Tzavalis test [40], which can be used when the number of time periods is small relative to the number of panels, we rejected the null hypothesis that the data generating process is non-stationary. Therefore, we included the lagged dependent variable in the model. We used cluster-robust standard errors to account for clustering that might occur at the network level. NC-CPESN pharmacies are grouped into regional networks by CCNC and may receive different levels and quality of implementation support across networks. All pharmacies received standardized training on how to document a CMR and how to use the documentation system that was required by NC-CPESN; however, the amount and type of technical assistance to support implementation of NC-CPESN (e.g., how to deliver a CMR) varied across networks [41]. Because the amount of missing data in both equations of the model was less than 10% (8.0 and 5.8%, respectively), we addressed missingness using complete case analysis. To test whether missingness might be correlated with the dependent variable, we compared the proportion of implementers and non-implementers between survey respondents and non-respondents and did not find significant differences (${\mathcal{X}}^2=2.27$, *p* = 0.132). We conducted the analyses using Stata version 13.0 (College Station, TX).

## Results

Of the 191 pharmacies in our sample, 113 (59.16%) were implementers. Pharmacies that successfully implemented a CMR had a significantly higher mean implementation climate (11.81 vs. 3.55, *p* < 0.001) and innovation-values fit (13.55 vs. 11.06, *p* < 0.001) scores (Table 1). In terms of patient needs and resources, implementing pharmacies were significantly more likely to participate in the 340B Drug Pricing Program (69.12 vs. 30.88%, *p* = 0.024) and have a higher proportion of high-risk patients (0.42 vs. 0.36, *p* = 0.004). For available resources, implementing pharmacies were more likely to have a clinical pharmacist (86.49 vs. 13.51%, *p* < 0.001) and either a pharmacy student or resident on staff (92.86 vs. 7.14%, *p* < 0.001). Implementing pharmacies had more experience with NC-CPESN (34.37 vs. 27.05 months, *p* < 0.001) and had a higher proportion of CMRs performed among high-risk patients in the previous quarter (0.03 vs. 0.00, *p* < 0.001). For structural characteristics of pharmacies, we did not find any significant differences between implementers and non-implementers.

**Table 1:** Descriptive statistics of community pharmacies participating in NC-CPESN

Characteristics	Implementers (*n* = 113) Mean (SD) or %	Non-implementers (*n* = 78) Mean (SD) or %	Total (*n* = 191) Mean (SD) or %	Range
Key independent variables				
Implementation climate	11.81 (3.0252)	3.55 (3.064)***	8.37 (5.087)	0–16
Innovation-values fit	13.55 (2.0218)	11.06 (3.99)***	12.51 (3.231)	0–16
Patient needs and resources				
Rural location	57.78	42.22	23.56	0–1
Clinical factors	31.94 (29.78)	39.63 (29.40)	35.08 (29.8)	1–100
Social factors	44.07 (30.8)	46.36 (33.17)	45.01 (31.8)	1–100
340B participation	69.12	30.88*	36.76	0–1
Proportion of high-risk patients	0.42 (0.14)	0.36 (0.18)**	0.40 (0.16)	0–0.87
Available resources				
Presence of a clinical pharmacist	86.49	13.51***	19.37	0–1
Total number of staff	12.83 (6.464)	11.53 (8.827)	12.30 (7.525)	1–40
Presence of pharmacy student or resident	92.86	7.14***	21.99	0–1
Access to knowledge about the intervention				
Amount of experience with NC-CPESN (months)	34.37 (7.0546)	27.05 (7.96)***	31.38 (8.249)	12.1–44.7
Past performance with NC-CPESN	0.03 (0.04)	0.00 (0.00)**	0.02 (0.0)	0–0.31
Participation in Medicare Part D MTM	67.27	32.73***	86.39	0–1
Structural characteristics				
Independent pharmacy	57.83	42.17	43.46	0–1
Low prescription volume	56.06	43.94	34.55	0–1
Established pharmacy	45.13	30.77	39.27	0–1

Significance of *t* tests or Pearson’s chi-square tests comparing implementers to non-implementers: *p < 0.05, **p < 0.01, ***p < 0.001

### Exploratory factor analysis

All pairwise correlations among the items in the implementation climate and innovation-values scales were greater than 0.30, indicating there was sufficient correlation for factor analysis (Table 2). Further, none of the pairwise correlations exceeded > 0.80, indicating that high multicollinearity was not a problem. The Bartlett’s test of sphericity was significant for the implementation climate (${\mathcal{X}}^2=1975.43,p<0.001\Big)$ and the innovation-values fit scale (${\mathcal{X}}^2=1077.83,p<0.001\Big)$. Therefore, we rejected the null hypothesis that either matrix was an identity matrix. The KMO statistic for implementation climate and innovation-values fit scales was 0.773 and 0.818, respectively, which are within an acceptable range to support factor analysis (greater than 0.60) [42].

**Table 2:** Correlation matrix for the implementation climate and innovation-values fit scales

Item	Implementation climate				Item	Innovation-values fit			
	1	2	3	4		1	2	3	4
1 Support—time	1.000				1 Professional values—advances field of pharmacy	1.000			
2 Support—resources	0.697	1.000			2 Organizational values—best care for patients	0.640	1.000		
3 Expectation	0.573	0.636	1.000		3 Organizational values—improves patient outcomes	0.661	0.677	1.000	
4 Reward	0.486	0.531	0.493	1.000	4 Professional values—what pharmacies should be doing	0.730	0.638	0.645	1.000

Factor loadings produced from the principal component analysis (Table 3) suggest that survey items measuring implementation climate or innovation-values fit load onto two distinct factors. For each set of items, only one factor had an eigenvalue exceeding 1.0 (implementation climate, largest EV = 2.77; innovation-values fit, largest EV = 3.35), and these eigenvalues were greater than eigenvalues from a randomly generated data set, suggesting one factor should be extracted for each set of items. The total amount of variance in the items explained by the two extracted factors was 79.27% for implementation climate and 83.63% for innovation-values fit. There were several items that had double factor loadings (e.g., loaded onto more than one factor) (Table 3); however, based on our decision rules as well as our theory, we retained one extracted factor for each set of items. Cronbach’s coefficient alpha was 0.845 for the implementation climate scale and 0.833 for the innovation-values scale, suggesting the items have “very good” internal consistency [43].

**Table 3:** Factor loadings from the rotated factor structure matrix for implementation climate and innovation-values fit scales

Implementation climate items	Factors			
	1	2	3	4
[Support—time] Our pharmacy allocates sufficient time to delivering enhanced pharmacy services.	*0.523*	− 0.293	− 0.396	**0.644**
[Support—resources] Our pharmacy devotes adequate resources to implementing enhanced pharmacy services.	*0.543*	− 0.296	− 0.218	− 0.055
[Expectation] In our pharmacy, we are expected to participate in the delivery of enhanced pharmacy services.	*0.487*	− 0.037	0.565	0.114
[Reward] In our pharmacy, individuals receive recognition for participating in the delivery of enhanced pharmacy services.	*0.442*	0.110	− 0.216	0.040
Innovation-values fit items	Factors			
	1	2	3	4
[Professional values] Delivering enhanced pharmacy services is what pharmacies should be doing.	*0.498*	− 0.224	0.251	− 0.232
[Organizational values] Delivering enhanced pharmacy services is consistent with providing the best care possible for our patients.	*0.501*	− 0.068	− 0.128	0.369
[Organizational values] Delivering enhanced pharmacy services is important for improving health outcomes for our patient population.	*0.506*	**0.406**	− 0.308	− 0.096
[Professional values] Delivering enhanced pharmacy services is important for advancing the field of pharmacy.	*0.495*	0.185	0.295	0.371

Factor loadings in boldface indicate double loading on two or more factors. Factor loadings in italics indicate the factor on which the item was placed

### Hurdle regression: equation 1

#### Hypothesis 1

The first equation of the hurdle regression indicated that a one-unit increase in the implementation climate score increased the probability of NC-CPESN implementation by 2.65 percentage points holding all else constant (*p* < 0.001) (Table 4). The predicted probability of NC-CPESN implementation for pharmacies with the median implementation climate score (9.14) was 0.66 compared to 0.84 for pharmacies with an implementation climate score at the 75th percentile (12.50).

**Table 4:** Parameter estimates from hurdle regression of NC-CPESN implementation and program reach of NC-CPESN implementation

Characteristics	Equation 1: binary (implementation) AME^a,b^ (SE)	Equation 2: positives (program reach) AME^a^ (SE)
Key independent variables		
Implementation climate^d^	2.65 (1.85 × 10^3^)^c^***	5.05 (1.5)**
Innovation-values fit^d^	2.17 (1.041 × 10^2^)*	11.79 (3.170)***
Patient needs and resources		
Rural location	−0.77 (0.016)	− 12.81 (4.658)**
Clinical factors	−0.04 (3 × 10^4^)	− 0.14 (0.11)
Social factors	−0.06 (3 × 10^4^)	− 0.10 (0.10)
340B participation	5.70 (3.50 × 10^2^)*	12.80 (5.760)*
Proportion of high-risk patients	0.00 (0.00)*	–
Log of high-risk patients	–	(exposure)
Available resources		
Presence of a clinical pharmacist	9.86 (4.75 × 10^2^)*	32.33 (10.670)***
Total number of staff	− 0.31 (2.6 × 10^3^)	− 1.98 (0.550)***
Presence of pharmacy student or resident	6.86 (6.37 × 10^2^)	14.55 (7.273)
Access to knowledge about the intervention		
Amount of experience with NC-CPESN (months)	0.43 (1.3 × 10^3^)**	1.57 (0.610)***
Past performance with NC-CPESN	0.46 (1.3 × 10^2^)***	0.10 (0.031)***
Participation in Medicare Part D MTM	18.73 (6.246 × 10^2^)**	28.05 (13.83)*
Structural characteristics		
Independent pharmacy	4.14 (2.02 × 10^2^)*	0.43 (5.6)
Low prescription volume	1.08 (0.032)	7.23 (7.21)
Established pharmacy	2.02 (0.015)	4.14 (7.46)
Alpha	–	0.56 (7.08 × 10^2^)**
Constant	− 21.04 (4.79)***	− 14.03 (1.383)***
Observations	180	104

Significance of hurdle regression: *p < 0.05, **p < 0.01, ***p < 0.001

^a^AME, average marginal effect

^b^Effect sizes for the stage 1 model are in percentage points; for example, 9.86 for presence of clinical pharmacist indicates that the probability of implementing NC-CPESN was 9.86 percentage points higher for pharmacies that have a clinical pharmacist

^c^Any standard errors that were carried out to the ten-thousandths place value or smaller are represented in scientific notation

^d^Equation 1 and 2 include an interaction term (implementation climate*innovation-values fit), which is represented in the AME of implementation climate and innovation-values fit

#### Hypothesis 2

Similarly, an increase in innovation-values fit score increased the probability of NC-CPESN implementation by 2.17 percentage points (*p* = 0.037). The predicted probability of NC-CPESN implementation for pharmacies with the median innovation-values score (13.07) was 0.61 compared to 0.66 for pharmacies with an implementation climate score at the 75th percentile (14.68).

#### Hypothesis 3

**Fig. 2:**
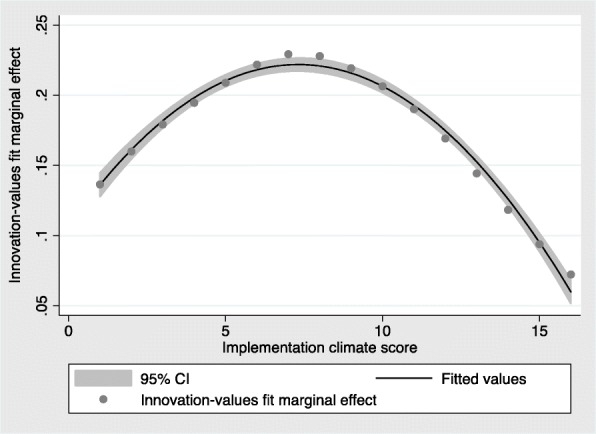
Plot of marginal effect of innovation-values fit and implementation climate score for Equation 1

The marginal effect of innovation-values fit on the probability of NC-CPESN implementation increased as implementation climate score increased. The marginal effect began to decline at an implementation score of 8 (Fig. 2).

#### Hypothesis 4

No significant differences in the probability of NC-CPESN implementation was found based on patients’ needs and resources. For available resources, the probability of implementing NC-CPESN was 9.86 percentage points higher for pharmacies that had a clinical pharmacist (*p* = 0.038). In terms of access to knowledge about the intervention and available resources, amount of experience with NC-CPESN (*p* = 0.004), past performance with NC-CPESN (*p* < 0.001), and participation in Medicare Part D MTM (*p* = 0.003) were each positively associated with the probability of implementing NC-CPESN. Within structural characteristics, the probability of implementing NC-CPESN was 4.14 percentage points higher among independently owned pharmacies (*p* = 0.041).

### Hurdle regression: equation 2

#### Hypothesis 1

Findings from the second equation of the hurdle regression indicated that a one-unit increase an implementation climate score was associated with a 5.05 increase in implementation of CMRs per high-risk patients holding all else constant (*p* = 0.001). The predicted number of CMRs per high-risk patients for pharmacies with the median implementation climate score (9.14) was 16.21 compared to 28.10 for pharmacies with an implementation climate score at the 75th percentile (12.50).

#### Hypothesis 2

Similarly, implementation of CMRs per high-risk patients was positively associated with innovation-values fit score (*p* < 0.001). The predicted number of CMRs per high-risk patients for pharmacies with the median innovation-values score (13.07) was 32.09 compared to 59.36 for pharmacies with an implementation climate score at the 75th percentile (14.68).

#### Hypothesis 3

**Fig. 3:**
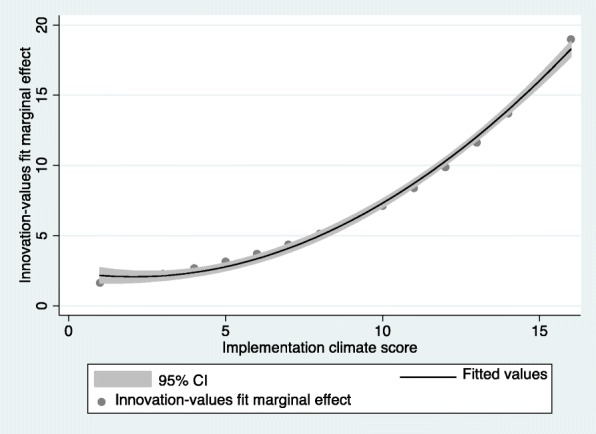
Plot of marginal effect of innovation-values fit and implementation climate score for Equation 2

The marginal effect of innovation-values fit on the number of CMRs per high-risk patients increased as implementation climate score increased (Fig. 3).

#### Hypothesis 4

In terms of patients’ needs and resources, pharmacies located in rural locations were associated with lower implementation of CMRs per high-risk patients (*p* = 0.006). Conversely, pharmacies that participate in the 340B Drug Pricing Program were associated with higher implementation (*p* = 0.026). For available resources, pharmacies with a clinical pharmacist were associated with higher implementation (*p* = 0.002). However, an increase in total staff was associated with a 1.98 decrease in implementation of CMRs per high-risk patients (*p* < 0.001). For available resources, implementation of CMRs per high-risk patients was positively associated with experience with NC-CPESN (*p* = 0.004), past performance with NC-CPESN (*p* < 0.001), and participation in Medicare Part D MTM (*p* = 0.003). No significant differences in implementation were found based on structural characteristics.

## Discussion

In this study, we used the organizational theory of innovation implementation effectiveness [18–20] to test organizational factors that influence implementation effectiveness of a community pharmacy medication management intervention. Consistent with our hypothesis, we found that key constructs from this theory, such as implementation climate and innovation-values fit, were positively associated with implementation and program reach of NC-CPESN. To our knowledge, only one other quantitative study has examined the relationship between implementation climate and implementation effectiveness in healthcare [31], and no other study in healthcare has explored the direct and indirect effects of innovation-values fit on implementation effectiveness. Contrary to our hypotheses of contextual factors, only certain factors, such as having a clinical pharmacist on staff, participation in Medicare Part D MTM, or 340B Drug Pricing Program, predicted both implementation and program reach. We were also surprised that 40.8% of the community pharmacies participating in the study did not have implementation activity within the study period. We describe potential reasons for this below.

We hypothesized that implementation climate and innovation-values fit would be positively and directly associated with implementation effectiveness, which was supported by our findings. These findings suggest that implementation climate and innovation-values fit were useful measures for predicting implementation and program reach. Further studies are needed to test whether these measures are predictive of implementation effectiveness across a wider variety of community pharmacy medication management programs. For example, we learned from the previous qualitative work that NC-CPESN community pharmacy staff worked collaboratively to implement CMRs. For other medication management programs, organizations may rely on a single staff member to deliver the intervention. In such cases, individual-referenced measures of implementation climate [32] may be more valid than group-referenced items.

The study results also supported the hypothesis that innovation-values fit moderates the effect of implementation climate on implementation effectiveness, indicating that implementation climate and innovation-values fit work in concert. Our findings also suggest that innovation-values fit may have a greater effect on implementation climate at lower levels of implementation climate and that the effect may diminish at higher levels of implementation climate (Fig. 2). Further research is needed to establish whether there are diminishing returns to the effect of innovation-values fit on implementation climate and whether the relationship depends on the outcome of interest, i.e., presence of implementation activity versus level of implementation activity (e.g., program reach). Additionally, future research is needed to determine what factors are positively associated with implementation climate and innovation-values fit in pharmacy medication management programs. For example, the organizational theory of innovation implementation effectiveness [18–20] maintains that management support is an antecedent of implementation climate, but there has been little quantitative research on how to operationalize the construct of management support. Recently, researchers have developed a measure for implementation leadership to assess which leadership qualities are correlated with successful implementation [44]. Future studies could assess whether implementation leadership is associated with implementation climate. This has practical importance because identifying the leadership behaviors and traits associated with effective implementation could provide guidance to pharmacy leaders on how to develop a supportive climate for medication management program implementation.

Contrary to our hypotheses, we found that only certain aspects of the organizational context affected both implementation and program reach. For example, none of the structural characteristics (e.g., pharmacy type, established pharmacy) were significantly associated with both implementation and program reach. Additional research is needed to determine whether there are other structural characteristics that may be associated with successful implementation of pharmacy medication management programs. Consistent with our hypotheses, access to knowledge about the intervention (e.g. participation in Medicare Part D MTM), patient needs and resources (e.g., proportion of high-risk patients, participation in 340B Drug Pricing Program), and availability of certain resources (e.g., clinical pharmacist) positively affect implementation effectiveness. Prior theory suggests that establishing an implementation climate for one intervention may help facilitate implementation climate for a similar intervention [29]. It is possible that community pharmacies use similar strategies to support MTM and medication management services implementation (e.g., staff training on motivational interviewing)—explaining the positive association between Medicare Part D MTM and NC-CPESN implementation. Future studies should use qualitative methods to explore the implementation strategies that community pharmacies establish to foster a climate for medication management services and whether these strategies facilitate implementation of similar interventions. Such studies could be used to develop implementation guidance to support community pharmacies participating in multiple medication management programs simultaneously, which may increase as pharmacy participation in alternative payment models grows. We also found that having a clinical pharmacist on staff was an important predictor of implementation effectiveness. However, clinical pharmacists’ roles and the amount of time available for clinical services can vary widely across community pharmacies [34, 35]. Future qualitative studies are needed to describe how community pharmacies define the job roles of clinical pharmacists, how much time clinical pharmacists are given for non-dispensing activities, and whether such differences are perceived to impact the effectiveness of clinical pharmacists.

Unexpectedly, we found that about 40% of the pharmacies participating in this study did not implement a CMR during the study period. From a recent qualitative study we conducted, we found that many of the pharmacies in the NC-CPESN program struggled not necessarily with conducting a CMR but with documenting a CMR [41]. Most pharmacies indicated that they received sufficient training on the documentation system but struggled with finding the time to document the CMR and found the process to be burdensome. Some pharmacies described having to hire additional staff to assist with documentation or having staff work overtime to keep up with documentation. To address this need, CCNC has updated the templates for CMR documentation and made improvements to the documentation system itself [45]. Future studies are needed to test whether the new system reduces the amount of time needed for documentation and improves NC-CPESN pharmacies ability to document CMRs. Such research would have applicability not only for NC-CPESN but also for other programs that require documentation of medication management services, such as the Medicare Part D MTM program.

### Limitations

This study had several limitations. First, since we measured implementation climate, innovation-values fit, and implementation effectiveness at the same time, we cannot establish the causal order. Second, the generalizability of our findings is limited by: (1) only having data at one time point; (2) conducting the study in NC, the first CPESN organization. Future studies are needed to examine implementation of medication management programs over time and across settings. Third, our measures of implementation effectiveness, implementation and program reach among high-risk patients, are limited in scope and do not assess other important aspects of implementation effectiveness such as fidelity of CMR delivery. Future studies are needed to establish additional measures of implementation effectiveness (e.g., conducting site observations to measure CMR fidelity). Finally, we did not measure other determinants of implementation effectiveness including the presence of an innovation champion or variability in implementation climate perceptions [18–20]. Future studies should develop and test these measures in pharmacy medication management programs.

## Conclusions

As more state Medicaid programs adopt pharmacist-led medication management programs, it is important to identify what organizational determinants promote effective implementation of these programs. Our study supported the use of the organizational theory of innovation implementation effectiveness to identify organizational determinants that are associated with effective implementation (e.g., implementation climate and innovation-values fit) [18–20]. Unlike broader environmental factors or structural characteristics (e.g., pharmacy type), implementation climate and innovation-values fit are modifiable factors and can be targeted through intervention—a finding that is important for community pharmacy practice. Additional research is needed to determine what implementation strategies can be used by community pharmacy leaders and practitioners to develop a positive implementation climate and innovation-values fit for medication management programs.

## Abbreviations

AME: Average marginal effect
CCNC: Community Care of North Carolina
CMR: Comprehensive medication review
CPESN^SM^: Community Pharmacy Enhanced Services Network program
KMO: Kaiser-Meyer-Olkin
MTM: Medication therapy management
